# Assessing the utility of statistical adjustments for imperfect detection in tropical conservation science

**DOI:** 10.1111/1365-2664.12272

**Published:** 2014-06-02

**Authors:** Cristina Banks‐Leite, Renata Pardini, Danilo Boscolo, Camila Righetto Cassano, Thomas Püttker, Camila Santos Barros, Jos Barlow

**Affiliations:** ^1^ Grand Challenges in Ecosystems and the Environment Department of Life Sciences Imperial College London Silwood Park Campus Ascot SL5 7PY UK; ^2^ Departmento de Ecologia Instituto de Biociências Universidade de São Paulo Rua do Matão 101, trav. 14 São Paulo SP 05508‐090 Brazil; ^3^ Departmento de Zoologia Instituto de Biociências Universidade de São Paulo Rua do Matão 101, trav. 14 São Paulo SP 05508‐090 Brazil; ^4^ Departamento de Biologia Faculdade de Filosofia Ciências e Letras de Ribeirão Preto Universidade de São Paulo ‐ USP Av. Bandeirantes 3900 Ribeirão Preto 14040‐901 Brazil; ^5^ Departamento de Ciências Biológicas Universidade Estadual de Santa Cruz Campus Prof. Soane Nazaré de Andrade Km 16 ‐ Rodovia Jorge Amado Ilhéus BA 45662‐900 Brazil; ^6^ Lancaster Environment Centre Lancaster University Lancaster LA1 4YQ UK; ^7^ Museu Paraense Emílio Goeldi Av. Magalhães Barata 376 Belém Pará CEP 66040‐170 Brazil

**Keywords:** biodiversity conservation, capture–recapture models, detectability, detection probability, imperfect detection, monitoring, occupancy models, species richness

## Abstract

1. In recent years, there has been a fast development of models that adjust for imperfect detection. These models have revolutionized the analysis of field data, and their use has repeatedly demonstrated the importance of sampling design and data quality. There are, however, several practical limitations associated with the use of detectability models which restrict their relevance to tropical conservation science.
2. We outline the main advantages of detectability models, before examining their limitations associated with their applicability to the analysis of tropical communities, rare species and large‐scale data sets. Finally, we discuss whether detection probability needs to be controlled before and/or after data collection.
3. Models that adjust for imperfect detection allow ecologists to assess data quality by estimating uncertainty and to obtain adjusted ecological estimates of populations and communities. Importantly, these models have allowed informed decisions to be made about the conservation and management of target species.
4. Data requirements for obtaining unadjusted estimates are substantially lower than for detectability‐adjusted estimates, which require relatively high detection/recapture probabilities and a number of repeated surveys at each location. These requirements can be difficult to meet in large‐scale environmental studies where high levels of spatial replication are needed, or in the tropics where communities are composed of many naturally rare species. However, while imperfect detection can only be adjusted statistically, covariates of detection probability can also be controlled through study design. Using three study cases where we controlled for covariates of detection probability through sampling design, we show that the variation in unadjusted ecological estimates from nearly 100 species was qualitatively the same as that obtained from adjusted estimates. Finally, we discuss that the decision as to whether one should control for covariates of detection probability through study design or statistical analyses should be dependent on study objectives.
5. *Synthesis and applications*. Models that adjust for imperfect detection are an important part of an ecologist's toolkit, but they should not be uniformly adopted in all studies. Ecologists should never let the constraints of models dictate which questions should be pursued or how the data should be analysed, and detectability models are no exception. We argue for pluralism in scientific methods, particularly where cost‐effective applied ecological science is needed to inform conservation policy at a range of different scales and in many different systems.

## Abstract 2

Models that adjust for imperfect detection are an important part of an ecologist's toolkit, but they should not be uniformly adopted in all studies. Ecologists should never let the constraints of models dictate which questions should be pursued or how the data should be analysed, and detectability models are no exception. We argue for pluralism in scientific methods, particularly where cost‐effective applied ecological science is needed to inform conservation policy at a range of different scales and in many different systems.

## Introduction

Imperfect detection is a problem common to all ecological studies, and researchers should always try to minimize the effects of covariates on detection probability. While in the past the influence of such covariates has been mostly controlled through study design, the use of models that estimate and control for detection probability has become increasingly common in ecology and conservation science (Boulinier *et al*. 1998; Gu & Swihart 2004; Kéry & Schmid 2004; Gibson 2011; Archaux, Henry & Gimenez 2012). Given the rapid growth in the use of these statistical methods, we believe it is timely to briefly review the main known advantages of models that control for imperfect detection and compare these with a novel assessment of their practical limitations. We here intend to do so from the perspective of tropical conservation science, where cost‐effective research is urgently needed to inform robust management (Metzger 2009; Ferreira *et al*. 2012).

This review is structured in the following way: (1) we briefly introduce some of the terminology and concepts related to imperfect detection and review the major benefits of models that adjust for detection probability. (2) We assess whether these analyses are applicable to all data sets and objectives, discussing some of the limitations of applying these models to rare species, species‐rich communities and studies requiring many spatial replicates. (3) We examine whether the use of statistics is more powerful than controlling for the effects of covariates on detection probability through a carefully planned study design. Our evaluation is based on field data from three different studies and focusses on models that are used for obtaining adjusted estimates of occupancy, abundance or species richness. Finally, we discuss when to control for covariates of detection probability through study design or through statistical analyses.

It is not our intention to discourage the use of models that adjust for imperfect detection: every analysis or method has advantages and disadvantages, and researchers should decide what is best for answering each particular question. We here call for pluralism in one's choice of scientific methods. As the mathematical ecologist Gauch (1982) once wrote: ‘To say that one research approach is better, in general, than another is of equivalent mentality to saying that a pH meter is more powerful than a microscope’.

## The uses of models that adjust for imperfect detection

For decades, researchers have used models that take into account the capture history of individuals of targeted species to estimate abundance, survival and other demographic rates (Otis *et al*. 1978; Lebreton *et al*. 1992). More recently, these models have been extended to provide estimates of occupancy, species richness and relative abundance that are adjusted for imperfect detection (Boulinier *et al*. 1998; Mackenzie & Kendall 2002; Mackenzie *et al*. 2002; Royle & Nichols 2003).

Imperfect detection occurs when a species or individual is present but is not detected (i.e. when detection probability, or *P*, is <1), and if ignored, imperfect detection leads to an underestimate of individual or species occurrence and to a potential bias in results obtained from ecological studies (Mackenzie *et al*. 2006). Estimates that are adjusted for detection probability, or *P*‐adjusted estimates, have become very popular as they can also control for the influence of covariates of detection probability, such as variation in climatic conditions over time, environmental variables among sampling sites, behavioural differences among individuals/species or variation in density across species. Another major benefit of these models is that they provide an estimate of the uncertainty present in the observation process. Failure to estimate and deal with uncertainty can lead to poor conservation and management decisions (Regan *et al*. 2005).

The use of models that adjust for imperfect detection, or detectability models, has brought much needed attention to the importance of study design and data quality. Moreover, they have advanced the field of population ecology and allowed informed decisions to be made about the conservation and management of target species, such as salamanders in the Great Smoky National Park, fritillary butterflies in the Swiss Alps and tigers in Myanmar (Bailey, Simons & Pollock 2004; Mackenzie *et al*. 2006; Cozzi, Mueller & Krauss 2008; Lynam *et al*. 2009; Rotella *et al*. 2009; Regan, Chades & Possingham 2011).

## The applicability of models that adjust for imperfect detection to tropical conservation science

### Analysing rare species

Ecological communities in the tropics are composed of many naturally rare species (Hubbell 2001), which complicates the use of models that control for imperfect detection. The Biological Dynamics of Forest Fragments Project (BDFFP), which is the largest and longest‐running project in the tropics (Ferraz *et al*. 2007; Laurance *et al*. 2011), provides an excellent illustration of these problems. During 13 years of avifaunal surveys, the project captured nearly 50 000 individuals from 178 bird species, an unusually large data set for tropical regions. Nonetheless, only 55 species were considered to be sufficiently detected to have their occupancy estimated (Ferraz *et al*. 2007); in other words, 70% of the species were too rare to be analysed with detectability models.

But what is a rare species? The definition of rarity often varies with study system and taxa (Rabinowitz 1981; Yu & Dobson 2000), and while it is undeniable that species that were only captured once or twice in such a large sample effort are rare, few would consider a species to be rare if it failed to occur in at least eight out of 11 sites, one of the criteria used by Ferraz *et al*. (2007). In fact, many of these supposedly rare species could easily have their presence/absence or number of captures/observations analysed under a generalized linear model (GLM) approach. Models that adjust for imperfect detection require an extra axis of information when compared to a GLM, as they also require a certain number of recaptures or observations over time. Thus, when a species or individual is poorly detected, detectability models may not converge to a solution even for a species occurring across 50% of the sites (Welsh, Lindenmayer & Donnelly 2013).

Paradoxically, this means that models that adjust for imperfect detection are most effective when a species or individual is commonly detected, yet in this situation there is less need to adjust for imperfect detection because the raw data already indicate if or when the species/individual is present. In theory, the main advantage of these analyses arises when the probability of detection is low, as the model gauges the uncertainty that exists in the data regarding whether the species or individual was absent or not detected, and then uses this measure to adjust the levels of occurrence or abundance. When detection probability is low, however, *P*‐adjusted estimates present very large confidence intervals (Welsh, Lindenmayer & Donnelly 2013), which means that analyses that adjust for imperfect detection fail to provide precise estimates when they are needed the most.

Another issue arising with the analyses of rare and poorly detected species is that their fitted probabilities are often found to be equal to one (Welsh, Lindenmayer & Donnelly 2013), a result that would generally suggest that the species is in effect widespread. In practice, this means that researchers are confronted with the following decision on how to interpret their results, given their choice of analyses. If unadjusted estimates are used, rare species could be interpreted as observed in only a few sites but, due to imperfect detection, they are likely to occur in other areas. If *P*‐adjusted estimates are used, rare species could instead be interpreted as occurring everywhere, although with great uncertainty. In areas where most species are rare and when there is a need to provide answers for multiple species, negative consequences for endangered species may potentially occur if the latter interpretation is chosen.

### Community‐level studies in species‐rich areas

It is obviously difficult to analyse a truly rare species at the population level regardless of whether imperfect detection is accounted for or not, and caution while interpreting the results is paramount in either case. However, rare species do not necessarily need to be analysed at the population level to provide ecological information. Unadjusted community metrics such as species richness, total abundance, community structure and composition can be calculated using data from all species, irrespective of their rarity, and these metrics can provide useful inferences about the conservation value and management of ecological systems (Barlow *et al*. 2007a; Pardini *et al*. 2010). This is particularly important for community‐level studies as these usually employ a generic technique to maximize the number of species detected, so their data set will always contain many rare or poorly detected species.

Community models adjust for imperfect detection at the species level, so often *P*‐adjusted community estimates can only be calculated from a small percentage of the community (e.g. 30–35%; Ferraz *et al*. 2007; Ruiz‐Gutiérrez, Zipkin & Dhondt 2010). Moreover, Ferraz *et al*. (2007) only obtained *P*‐adjusted estimates for 30% of species because these authors estimated species occupancy, which allows one to work with much coarser data than *P*‐adjusted estimates of abundance. It is doubtful whether it would be possible to calculate adjusted abundance estimates for more than a handful of species, even with the longest‐running surveys in the tropics. For those without access to such a large data set, fitting occupancy models is already a challenge. A search on the Web of Knowledge for ecological studies on the topic ‘occupancy’, combined with either ‘detectability’ or ‘detection probability’, revealed a total of 121 studies that were cited >10 times. Of these, 95% were either from the temperate zone (*n *=* *77), theoretical (*n *=* *11) or had focussed on a single tropical species (*n *=* *7, see Appendix S1 in Supporting Information). So, in the tropics, researchers wishing to use *P*‐adjusted measures of community are often restricted to focussing on small proportion of commonly detected species (Ferraz *et al*. 2007; Ruiz‐Gutiérrez, Zipkin & Dhondt 2010).

### Throwing out the baby with the bathwater: the risk of focussing on generalists

While it is certainly desirable to obtain *P*‐adjusted ecological estimates and a measure of uncertainty, detectability models may have important negative consequences when applied to community or species‐rich data. Species that are widespread and commonly observed are often generalists that tolerate a wide range of anthropogenic disturbance. For instance, we looked whether there is a correlation between commonness or rarity and species sensitivity to human disturbance using information from more than 3000 bird species from South America (Stotz *et al*. 1996). The trend was clear – among the species that are commonly observed, there were 403 species that are weakly sensitive to habitat disturbance, while only 54 species were highly sensitive (Table 1). Across rare birds, 96 species were highly sensitive, while only 10 had low sensitivity to habitat disturbance (Table 1). These results reinforce the notion that analysing only the most common species in a data set is likely to yield misleading results (Pardini *et al*. 2010; Banks‐Leite, Ewers & Metzger 2012). The effects of environmental changes are thus likely to be strongly underestimated if analyses are restricted to the commonly detected generalist species, leading studies to predict more optimistic conservation outcomes than the reality (Banks‐Leite, Ewers & Metzger 2012).

**Table 1:** Contingency table showing the number of Neotropical bird species represented in each category of sensitivity to human disturbances and abundance. Data were obtained from Stotz *et al*. (1996)

Abundance	Sensitivity		
	Low	Medium	High
Common	403	220	54
Fairly common	373	749	306
Uncommon	71	350	299
Rare	10	80	96

### The trade‐off between temporal and spatial replication

Accuracy and precision of *P*‐adjusted estimates is highly dependent on the number of repeated site‐specific surveys. MacKenzie & Royle (2005) provide a table of the number of surveys that should be conducted at each site given the probability of detection and true occupancy of the species. If the probability of detection is high (*P *≥* *0·7), the optimum number of surveys to conduct at each site is 2–3, but if the probability of detection is low (*P *=* *0·1), the suggested number of surveys to conduct at each site can be as high as 34 (MacKenzie & Royle 2005). Studies that do not perform such optimum number of surveys fail to fit occupancy models to data, resulting in highly variable outcomes and unstable fitted occupancy probabilities (Welsh, Lindenmayer & Donnelly 2013).

Although temporal replication (i.e. representation) is undoubtedly important (even in relatively aseasonal areas: Barlow *et al*. 2007b; Banks‐Leite *et al*. 2012), there is a trade‐off between the number of locations that can be surveyed and temporal repetitions per location due to logistical, expertise and financial constraints (Guillera‐Arroita, Ridout & Morgan 2010). For instance, in *Study 3* (see below), we sampled birds in 65 sites for an average of 5 days per site, and our results showed that the average detection probability across species was 0·12, and the average occupancy was found to be 0·47. According to MacKenzie & Royle (2005), the appropriate number of temporal repetitions for *Study 3* should have been 17–18 days per site. If we assume that the total budget for the project is fixed, increasing the number of repetitions from five to 17–18 days per site would have led to a reduction in the number of spatial replicates from 65 to 18, thus precluding our ability to address conservation‐relevant questions (Banks‐Leite *et al*. 2011; Banks‐Leite, Ewers & Metzger 2012; Lira *et al*. 2012). This example shows just how impractical it is to study the effects of environmental change by conducting a large‐scale study as well as a large number of surveys per site.

In many cases, it is questionable whether spatial resolution should be sacrificed over temporal replication to obtain improved estimates for only a limited number of species. For instance, habitat and climate change are the main drivers of biodiversity loss (Millennium Ecosystem Assessment 2005), but the effects of environmental change are not the same across regions or biomes. Instead, they are dependent on many factors, such as landscape context, original habitat structure, land use type, spatial scale of interest, altitude, latitude, among many other variables (Gardner *et al*. 2009). Hence, the most pressing questions in conservation science require large‐scale investigations (Gardner *et al*. 2009; Ferreira *et al*. 2012; Ramage *et al*. 2012). Unfortunately, this has not always been successfully achieved in ecological science, constraining our ability to gather a good understanding of these processes and to provide useful management recommendations (Ramage *et al*. 2012). Obtaining *P*‐adjusted estimates of occurrence for a few (generalist) species in a few sites is unlikely to guide the development of effective policies in such species‐rich environments.

To summarize, models that adjust for imperfect detection are likely to have low applicability to community‐level studies from the species‐rich tropics because a large number of species are rare. The consequences of such shortcomings are potentially dangerous if researchers ignore the large uncertainties associated with adjusted estimates obtained for species with low detection rates, or if community patterns are biased by the responses of a few common and generalist species. Moreover, if the focus of the study is on the effects of large spatial scale environmental change, models that adjust for imperfect detection are not always a viable or desirable option.

## The need for models that adjust for imperfect detection

### Imperfect detection needs to be controlled either ‘before or after’ data collection, and not ‘before and after’

Unadjusted estimates are often underestimates of the true abundance, occupancy or species richness. Such underestimation can have a strong influence on the quality of information used to guide management actions such as hunting quotas or endangerment listings (White 2005; Mackenzie *et al*. 2006). In this context, there is a clear importance of controlling for imperfect detection to obtain an estimate of species occupancy or abundance that is closer to what would be achieved with a census of the entire population. The aim of many ecological studies, however, is not to obtain an absolute ecological estimate for a given species; most often the aim is to investigate the effects and the interactions of environmental factors on populations and communities. This is similar to say that, in the case of a regression model, the interest lies in obtaining a measure of the strength and direction of an ecological effect rather than the intercept.

Although imperfect detection *per se* cannot be controlled by study design without conducting a census (i.e. the survey of every single individual or species), researchers have always used their knowledge on the study system to list potential covariates of detection probability and plan the sampling design to minimize their influence. This process usually requires a lot of effort in the field through rigorous standardization of methods across sampling units. Nonetheless, it is widely accepted in the literature that even a well‐planned study will not sufficiently control for confounding factors (e.g. Mackenzie *et al*. 2006), because unmeasured variables could still be affecting detection probability and leading to biased estimates of ecological responses to a given environmental factor. Ecologists are now being strongly encouraged to use *a posteriori* statistical models that adjust for detection probability irrespective of having planned a rigorous sampling design (Boulinier *et al*. 1998; Gu & Swihart 2004; Kéry & Schmid 2004; Gibson 2011; Archaux, Henry & Gimenez 2012).

Studies using *P*‐adjusted estimates found that species richness increases with area, that isolation is detrimental to biodiversity and that rain forest species prefer to forage inside forests (Ferraz *et al*. 2007; Ruiz‐Gutiérrez, Zipkin & Dhondt 2010; Boscolo & Metzger 2011; Püttker *et al*. 2011, 2013), which are the same findings and the same implications to conservation reported in studies that only controlled for covariates of detection probability through careful sampling design (Barlow *et al*. 2007a; Ewers, Thorpe & Didham 2007; Boscolo & Metzger 2009; Pardini *et al*. 2010; Banks‐Leite *et al*. 2011). Thus, it is still an open question to what extent estimates of ecological responses to environmental factors derived from *P*‐adjusted models differ from those derived from unadjusted models, when covariates of detection probability are purposefully controlled before data collection. We explored this question by reanalysing data on almost 100 species from three independent studies conducted in the Atlantic Forest of Brazil, where particular care was given during sampling design to control for covariates that could influence detection probability.

### Case studies: are *P*‐adjusted estimates different to unadjusted estimates when covariates of detection probability are controlled during data collection?

#### Study 1: large mammals in cacao agroforests

##### Main aim

To investigate how the use of agroforests by large mammals is affected by the record rate of domestic dogs.

##### Data and sampling design

The study was developed in cacao plantations located in an agroforestry mosaic in southern Bahia, Brazil. Data on native mammals and domestic dogs were collected with camera‐traps in 30 sites (see Fig. S1 in Appendix S1, Supporting Information). Two camera‐traps were placed in each site, one on the ground and one in the understorey (3–4 m above ground level). Data collection was conducted during four surveys of 3 months each, equally divided in summer and winter from 2007 to 2009. At each survey, sites were grouped into three blocks, and all 10 sites within a block were simultaneously sampled within 1 month, and all 30 sites were sampled within 3 months (Cassano, Barlow & Pardini 2014).

##### Analysis

We used single‐season occupancy models (Mackenzie *et al*. 2006) to estimate the effect of the record rate of domestic dogs on site occupancy (ψ) by mammal species. For each species, we constructed a set of five candidate models. All candidate models had ψ as a function of domestic dog record rate (number of days with records of domestic dogs divided by the number of sampling days) and detection probability (*P*) either constant or modelled as a function of one of the following survey variables: sampling effort (in trap‐days), block (1 to 3), season (winter × summer) and survey number (1 to 4). We used generalized linear models to investigate the influence of domestic dog record rate on unadjusted estimates of species occurrence. We used model selection based on the Akaike Information Criterion corrected for small samples (AICc) to compare the set of candidate occupancy models (Burnham & Anderson 2002).

##### Results

**Figure 1:**
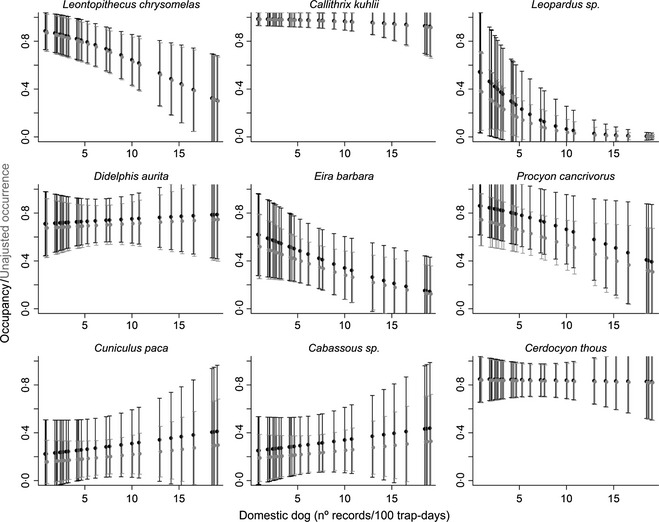
Predicted values (±CI) of the unadjusted estimates of occurrence and occupancy of 30 agroforests for nine large mammal species as a function of domestic dog capture rate. Values calculated using the best fit occupancy model for each species (see Appendix S2 and Tables S1 and S2 in Supporting Information).

We obtained 1694 records of 20 native mammals. However, we were able to estimate ψ and *P* just for nine species that were recorded in six or more sites. The influence of domestic dog on site occupancy differed among species (Fig. 1). For all analysed species, the set of plausible models included one or more covariates of detection probability (Table S1 in Supporting Information), showing that *P* was dependent on survey number (observed for six species), season (three species), sampling effort (two species) and block (two species). Average *P* across species was 0·47 (Table S2 in Supporting Information). Despite the strong influence of different covariates on detection probability, no significant difference was observed between modelled ψ and unadjusted occurrence, as 95% confidence intervals overlapped (Fig. 1) (Table S1 in Supporting Information).

### Study 2: small mammal population size in continuous forest

#### Main aim

To assess monthly variation in population size of four neotropical small mammals.

#### Data and sampling design

Small mammals were sampled in three trapping grids in a continuously forested landscape (Morro Grande Forest Reserve) in the State of São Paulo, Brazil. Each of the three grids encompassed 2 ha and consisted of 11 parallel 100‐m‐long lines, 20 m apart, with 11 trapping stations spaced every 10 m. In each trapping station, a Sherman trap was placed on the ground, and trapping stations of five alternated lines were additionally equipped with one 60‐l pitfall trap per station, connected to each other by a 50‐cm‐high plastic drift fence. Small mammals were captured simultaneously in the three grids during 21 monthly 5‐day trapping sessions from March 2008 to October 2009. Captured animals were marked and released in the respective trapping location. Trapping effort was 2640 trap nights per session, adding up to 55 440 trap nights in total. See Püttker *et al*. (2011, 2013) for further details.

#### Analyses

**Figure 2:**
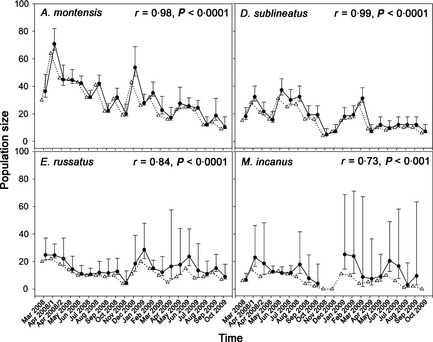
Monthly population sizes of three Atlantic Forest small mammals estimated by closed population estimates (circles, solid line, ±95% confidence intervals) and unadjusted estimate (MNKA; triangles, spotted line). Pearson's correlation coefficient (r) and probability of significance for each species are given in the upper right corner. For *Marmosops incanus*, abundance could not be estimated in November and December 2008 as well as in October 2009 due to low capture probabilities (see Appendix S2 and Tables S3, S4 and S5 in Supporting Information).

We estimated monthly abundance, probabilities of survival and capture and recapture probabilities of the four most abundant small mammals (Fig. 2). Estimates were obtained using Pollock robust design model in the program MARK (White & Burnham 1999), by pooling capture histories of the three trapping grids. Because our data sets did not allow for heavily parameterized models, the estimation of population size (N) was conditioned out of the likelihood using Huggins’ closed‐capture models within primary capture sessions (Huggins 1991). We formulated 10 candidate models differing in assumptions on survival probability *S*, capture probability and recapture probabilities (Table S3 in Supporting Information). In all models, capture probability and recapture probabilities were assumed constant within primary capture sessions. For each species, we used monthly population size estimates obtained by the most plausible model (lowest AICc) for comparison with unadjusted estimates. Unadjusted estimates were the number of individuals captured in each primary capture session. We compared abundance estimates between methods by Pearson's product–moment correlation.

#### Results

We obtained 4065 captures of 1380 individuals from 24 species, but only the four most common species were captured frequently enough to obtain *P*‐adjusted estimates of population size. Mean estimated population size differed among species, with *Akodon montensis* reaching highest abundance (mean ± SE estimated = 31·6 ± 3·2; number of individuals captured = 29·0 ± 3·0), followed by *Delomys sublineatus* (18·0 ± 2·1; 15·62 ± 1·7), *Euryoryzomys russatus* (15·7 ± 1·3; 11·7 ± 1·1) and *Marmosops incanus* (11·3 ± 1·7; 6·4 ± 1·0), showing that the rank abundance of these species remained the same with or without adjustments for imperfect detection. The most plausible model indicated a constant capture and recapture probability in only one species (*D. sublineatus*, Table S3 in Supporting Information), while in all other species the most plausible model indicated capture and recapture probabilities varying between capture sessions (Table S4 in Supporting Information). Estimated capture probabilities were below 0·2 in some months (Tables S3, S4 and S5 in Supporting Information), resulting in low precision of adjusted population size estimates (Fig. 2). Although monthly variation in population size obtained by unadjusted estimates differed occasionally from *P*‐adjusted estimates (i.e. months with low capture probabilities), number of individuals captured and estimated population sizes were highly correlated for the four species of small mammals (Fig. 2).

### Study 3: bird community in fragmented tropical landscapes

#### Main aim

To estimate how species richness varies among landscapes with different amounts of forest cover.

#### Data and sampling design

Field data were collected in the State of São Paulo, Brazil. Sampling was conducted in six 10000‐ha landscapes, three of which were fragmented but varied in the total amount of native rain forest (roughly 10, 30 and 50% of forest cover), while the remaining three had continuous forest cover, approximately 90% cover (Fig. S2 in Appendix S1, Supporting Information; Banks‐Leite *et al*. 2011). In each fragmented landscape, we sampled 17 to 19 forest patches ranging from 2 to 150 ha, and 12 sites in the continuous landscapes. Birds were sampled from 2001 to 2007, and mist nets were generally open from sunrise to sunset. All captured birds were marked and released in the vicinity. In total, we obtained over 7000 captures from 140 bird species with a sampling effort of 41000 net‐hours (each site was sampled from 3 to 9 times, mean effort per site 637 net‐hours, SD = 77).

#### Analysis

We estimated occupancy across the four forest cover treatments (10, 30, 50 and 90%) for all 84 species that occurred ≥ three sites, using a hierarchical community model developed by Zipkin, Dewan & Royle (2009). The predictor variable used for estimating occupancy (ψ) was landscape cover (categorical), and the predictor variables used for estimating *P* were forest patch size and amount of sampling effort per survey per site. Model parameters were estimated using a Bayesian analysis, with vague priors, carried out in WinBUGS (see model codes in Appendix S2, Supporting Information). At each forest cover treatment, *P*‐adjusted species richness was calculated as ∑ψ of all 84 species, and unadjusted species richness was calculated as ∑ (occupied sites/sites sampled) for all 84 species.

#### Results

**Figure 3:**
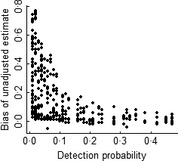
Bias of unadjusted estimate, defined as the absolute difference from *P*‐adjusted estimates (i.e. occupancy), increases at lower levels of detection probability (*P* < ~0·15). Data points represent species‐specific bias estimates calculated in each of the four forest cover treatments (10, 30, 50 and 90%), and thus, there are in total 336 data points (84 species × 4 forest cover treatments). Previous inspection of the data showed that the trend of increase in bias did not vary among landscapes (see Appendix S2 and Table S6 in Supporting Information).

The Bayesian *P*‐value of the model was estimated at 0·33, which suggests that the hierarchical model provided an adequate description of the data. Detection probability was significantly affected by sampling effort (β = 0·09, SD = 0·01) and patch area (β = 0·07, SD = 0·03), and species‐specific detection probabilities were in average *P *=* *0·12 (Table S6 in Supporting Information). The difference between ψ and unadjusted estimates of occurrence increased at lower values of detection probability (Fig. 3), which would suggest that unadjusted estimates are strongly biased. However, the overall pattern of variation in species occupancy was the same as the one observed for unadjusted occurrence (Table S6 in Supporting Information). In the landscapes with 10, 30, 50 and 90% forest cover, *P*‐adjusted species richness was 37·8, 36·6, 40·2 and 43·5, while unadjusted species richness was 25·0, 24·5, 26·3 and 28·8, respectively. In general, unadjusted and *P*‐adjusted species richness followed the same trend of variation as unadjusted species richness, but corrected by a factor of 1/0·66.

## Should covariates of detection probability be controlled before or after data collection?

Although we criticize the dogmatic use of models that adjust for imperfect detection, we strongly believe in the importance of controlling for covariates of detection probability in ecological studies. The similarity of results obtained from *P*‐adjusted and unadjusted estimates in the three studies above was only found because we attempted to control all known sources of bias and covariates of detection probability prior to data collection. Thus, covariates of detection probability must be controlled either before or after data collection, and the question becomes when should these covariates be controlled. In our opinion, the answer to this question depends on the objectives of a study.

### Controlling for covariates through statistics or after data collection

If the aim is to use ecological estimates for reserve or game management, pest control or any other use that requires robust absolute values or robust estimates of uncertainty for a single species, then it is sensible to use models that adjust for imperfect detection. First, unadjusted indices are almost always underestimates, and *P*‐adjusted estimates are likely to be one step closer to the true value. Secondly, specific objectives such as game management allow one to tailor data collection to maximize species detection, thus allowing the use of detectability models. Another study objective that likely requires the use of *P*‐adjusted estimates is when researchers are analysing data collected by many observers with different abilities, or data that were collated from several different sources (such as Tingley & Beissinger 2012).

There is the risk, however, that an overreliance on statistics for adjusting covariates of imperfect detection can also lead researchers to reduce their attention to the study design. The fact that *P*‐adjusted estimates are corrected for imperfect detection does not mean that they are correct. In fact, it has been noted that there are situations in which *P*‐adjusted estimates are no more reliable than unadjusted estimates (Welsh, Lindenmayer & Donnelly 2013). It is possible that the estimate was adjusted for some important covariates but not others, or the study design was so unbalanced that even the best of all statistical corrections will not suffice. In the same way that spurious correlations may affect the detection of effects on the response variable, researchers may also wrongly identify a spurious covariate of detection probability that could adjust detection probability in undesired ways. Data are only as good as the methods that were used to collect them and not all biases can be fixed statistically, particularly in heavily unbalanced study designs.

### Controlling for covariates through experimental design or before data collection

In other cases, it will be more sensible to control for covariates of detection probability before data collection. This includes studies aiming at estimating ecological responses to environmental factors (i.e. in which the absolute value of ecological estimates is not the focus), studies involving a large number of species, including rare species or species difficult to detect, studies assessing large spatial scales, snapshot studies or when several sources of bias must be controlled. Controlling for covariates of detection probability before data collection allows controlling for more covariates of detection probability than it is feasible to fit *a posteriori* in a model. For instance, for *Study* 3, we controlled for time of day, season, extreme weather, habitat, slope, altitude, vegetation structure, proximity to rivers, proximity to forest edge, matrix type, mist net brand and length and observer. It would not have been possible to include all these variables in a model without a very large number of replicates. Moreover, as both methods are not mutually exclusive, models that adjust for imperfect detection can be used in addition to a standardized protocol to obtain a measure of the influence of the covariates, *P*‐adjusted estimates and a measure of uncertainty for at least some species. For this reason, when possible, it is always wise to plan a study design to have an adequate number of repeated surveys, to ensure that models that adjust for imperfect detection can be used for at least a few species. Finally, it is important to point out that unadjusted estimates can also be dangerous for conservation and management, especially if researchers are not mindful that these are underestimates of the ecological estimates.

In summary, in the three studies, detection probability was always <1 and accordingly *P*‐adjusted estimates of occupancy, population size and species richness were usually higher than unadjusted estimates (Table S5 in Supporting Information) (Fig. 2). Nonetheless, the ecological responses to analysed factors and conservation implications did not differ between statistically adjusted and unadjusted models of occurrence/abundance. The consistency of these results contrasts to the many examples where unadjusted estimates provided different results to *P*‐adjusted estimates (e.g. Mackenzie *et al*. 2006; Tingley & Beissinger 2012). Such disparate outcomes could be attributed to the fact that the sampling design in the three case studies was planned to minimize the effects of covariates of detection probability, although it is difficult to ascertain exactly how much attention was given to study design in other examples where *P*‐adjusted estimates provide different results. Covariates of detection probability can bring a strong bias to results, and their effects can be controlled either before or after data collection. The decision on whether to control for covariates of detection probability is heavily dependent on the objectives of a study and its ecological context.

## Conclusion

There are advantages and disadvantages to the use of models that control for imperfect detection. While the advantages are appealing in many ecological contexts, we outline several important limitations of these models that are particularly relevant to tropical conservation science. In a review of the application of methods that control for imperfect detection on bird data, Johnson (2008) concluded: ‘At present, no method of adjusting bird count data appears to be effective for large‐scale, multi‐species monitoring surveys’. We agree with Johnson's statement and suggest that the blind enforcement of the use of such analyses could be detrimental to conservation science, especially if researchers allocate more effort towards temporal repetition rather than spatial coverage, or when a large part of a data set is discarded due to model constraints. We do not believe that hard‐won field data, often on rare specialist species, should be uniformly discarded to accord with statistical models.

Given a well‐thought out and balanced sampling design, we suggest that unadjusted estimates of single‐ and multiple‐species responses to ecological gradients can be just as robust as estimates that were *a posteriori* controlled for covariates of detection probability. We strongly believe that inferences derived from studies based on ecologist's detailed *a priori* knowledge of their system are likely to be more valuable than those resulting from a poorly designed study followed by *a posteriori* adjustments of detectability. Most importantly, our main message is that one should always use the best method available for the data on hand and for the goals to be achieved.

## Supporting information


**Appendix S1.** List of publications obtained from the *Web of Knowledge* in 2012.

**Appendix S2.** Model codes used to calculate species occupancy at the landscapes with 10, 30, 50 and 90 forest cover, while accounting for changes in detectability due to sampling effort and patch size.

**Table S1.** Model selection results for nine species of mammals (*Study 1*).
**Table S2.** Detection probabilities (*P*) and standard error for nine species of mammals (Study 1) estimated as a function of survey, and constant through time.
**Table S3.** Candidate model set for estimation of survival probability (S), capture probability (*P*) and recapture probability (c) (Study 2). Parameters are either constant (.), or dependent on the session (time‐dependent ‐ t).
**Table S4.** Model selection results for *Akodon montensis*,* Delomys sublineatus*,* Euryoryzomyys russatus* and *Marmosops incanus* (Study 2).
**Table S5.** Estimated capture probabilities (*P*) and recapture probabilities (c), lower and upper 95% confidence intervals based on most plausible model for *Akodon montensis*,* Delomys sublineatus*,* Euryoryzomyys russatus* and *Marmosops incanus* (Study 2). n.e. = not estimable due to low capture probabilities
**Table S6.** Bird species‐specific estimates of occupancy, standard deviation and unadjusted estimate of occurrence per forest cover treatment, and overall probability of detection (*Study 3*).

## References

[jpe12272-bib-0001] Archaux, F. , Henry, P.Y. & Gimenez, O. (2012) When can we ignore the problem of imperfect detection in comparative studies? Methods in Ecology and Evolution, 3, 188–194.

[jpe12272-bib-0002] Bailey, L.L. , Simons, T.R. & Pollock, K.H. (2004) Estimating site occupancy and species detection probability parameters for terrestrial salamanders. Ecological Applications, 14, 692–702.

[jpe12272-bib-0003] Banks‐Leite, C. , Ewers, R.M. & Metzger, J.P. (2012) Unraveling the drivers of community dissimilarity and species extinction in fragmented landscapes. Ecology, 93, 2560–2569. 23431587 10.1890/11-2054.1

[jpe12272-bib-0004] Banks‐Leite, C. , Ewers, R.M. , Kapos, V. , Martensen, A.C. & Metzger, J.P. (2011) Comparing species and measures of landscape structure as indicators of conservation importance. Journal of Applied Ecology, 48, 706–714.

[jpe12272-bib-0005] Banks‐Leite, C. , Ewers, R.M. , Pimentel, R.G. & Metzger, J.P. (2012) Decisions on temporal sampling protocol influence the detection of ecological patterns. Biotropica, 44, 378–385.

[jpe12272-bib-0006] Barlow, J. , Gardner, T.A. , Araujo, I.S. , Avila‐Pires, T.C. , Bonaldo, A.B. , Costa, J.E. *et al* (2007a) Quantifying the biodiversity value of tropical primary, secondary, and plantation forests. Proceedings of the National Academy of Sciences, 104, 18555–18560. 10.1073/pnas.0703333104 PMC2141815 18003934

[jpe12272-bib-0007] Barlow, J. , Overal, W.L. , Araujo, I.S. , Gardner, T.A. & Peres, C.A. (2007b) The value of primary, secondary and plantation forests for fruit‐feeding butterflies in the Brazilian Amazon. Journal of Applied Ecology, 44, 1001–1012.

[jpe12272-bib-0008] Boscolo, D. & Metzger, J.P. (2009) Is bird incidence in Atlantic forest fragments influenced by landscape patterns at multiple scales? Landscape Ecology, 24, 907–918.

[jpe12272-bib-0009] Boscolo, D. & Metzger, J.P. (2011) Isolation determines patterns of species presence in highly fragmented landscapes. Ecography, 34, 1018–1029.

[jpe12272-bib-0010] Boulinier, T. , Nichols, J.D. , Sauer, J.R. , Hines, J.E. & Pollock, K.H. (1998) Estimating species richness: the importance of heterogeneity in species detectability. Ecology, 79, 1018–1028.

[jpe12272-bib-0011] Burnham, K.P. & Anderson, D. (2002) Model Selection and Multimodel Inference: A Practical Information‐Theoretic Approach. Springer, Fort Collins.

[jpe12272-bib-0012] Cassano, C.R. , Barlow, J. & Pardini, R. (2014) Forest loss or management intensification? Identifying causes of mammal decline in cacao agroforests. Biological Conservation, 169, 14–22.

[jpe12272-bib-0013] Cozzi, G. , Mueller, C.B. & Krauss, J. (2008) How do local habitat management and landscape structure at different spatial scales affect fritillary butterfly distribution on fragmented wetlands? Landscape Ecology, 23, 269–283.

[jpe12272-bib-0014] Ewers, R.M. , Thorpe, S. & Didham, R.K. (2007) Synergistic interactions between edge and area effects in a heavily fragmented landscape. Ecology, 88, 96–106. 17489458 10.1890/0012-9658(2007)88[96:sibeaa]2.0.co;2

[jpe12272-bib-0015] Ferraz, G. , Nichols, J.D. , Hines, J.E. , Stouffer, P.C. , Bierregaard, R.O. Jr & Lovejoy, T.E. (2007) A large‐scale deforestation experiment: effects of patch area and isolation on Amazon birds. Science, 315, 238–241. 17218527 10.1126/science.1133097

[jpe12272-bib-0016] Ferreira, J. , Pardini, R. , Metzger, J.P. , Fonseca, C.R. , Pompeu, P.S. , Sparovek, G. & Louzada, J. (2012) Towards environmentally sustainable agriculture in Brazil: challenges and opportunities for applied ecological research. Journal of Applied Ecology, 49, 535–541.

[jpe12272-bib-0017] Gardner, T.A. , Barlow, J. , Chazdon, R.L. , Ewers, R.M. , Harvey, C.A. , Peres, C.A. & Sodhi, N.S. (2009) Prospects for tropical forest biodiversity in a human‐modified world. Ecology Letters, 12, 561–582. 19504750 10.1111/j.1461-0248.2009.01294.x

[jpe12272-bib-0018] Gauch, H.G. (1982) Multivariate Analysis in Community Ecology. Cambridge University Press, Cambridge.

[jpe12272-bib-0019] Gibson, L.A. (2011) The importance of incorporating imperfect detection in biodiversity assessments: a case study of small mammals in an Australian region. Diversity and Distributions, 17, 613–623.

[jpe12272-bib-0020] Gu, W.D. & Swihart, R.K. (2004) Absent or undetected? Effects of non‐detection of species occurrence on wildlife‐habitat models. Biological Conservation, 116, 195–203.

[jpe12272-bib-0021] Guillera‐Arroita, G. , Ridout, M.S. & Morgan, B.J.T. (2010) Design of occupancy studies with imperfect detection. Methods in Ecology and Evolution, 1, 131–139.

[jpe12272-bib-0022] Hubbell, S.P. (2001) The unified Neutral Theory of Biodiversity and Biogeography. Princeton University Press, Princeton.

[jpe12272-bib-0023] Huggins, R.M. (1991) Some practical aspects of a conditional likelihood approach to capture experiments. Biometrics, 47, 725–732.

[jpe12272-bib-0024] Johnson, D.H. (2008) In defense of indices: the case of bird surveys. Journal of Wildlife Management, 72, 857–868.

[jpe12272-bib-0025] Kéry, M. & Schmid, H. (2004) Monitoring programs need to take into account imperfect species detectability. Basic and Applied Ecology, 5, 65–73.

[jpe12272-bib-0026] Laurance, W.F. , Camargo, J.L.C. , Luizão, R.C.C. , Laurance, S.G. , Pimm, S.L. , Bruna, E.M. *et al* (2011) The fate of Amazonian forest fragments: a 32‐year investigation. Biological Conservation, 144, 56–67.

[jpe12272-bib-0027] Lebreton, J.D. , Burnham, K.P. , Clobert, J. & Anderson, D.R. (1992) Modeling survival and testing biological hypotheses using marked animals: a unified approach with case studies. Ecological Monographs, 62, 67–118.

[jpe12272-bib-0028] Lira, P.K. , Ewers, R.M. , Banks‐Leite, C. , Pardini, R. & Metzger, J.P. (2012) Evaluating the legacy of landscape history: extinction debt and species credit in bird and small mammal assemblages in the Brazilian Atlantic Forest. Journal of Applied Ecology, 49, 1325–1333.

[jpe12272-bib-0029] Lynam, A.J. , Rabinowitz, A. , Myint, T. , Maung, M. , Latt, K.T. & Po, S.H. (2009) Estimating abundance with sparse data: tigers in northern Myanmar. Population Ecology, 51, 115–121.

[jpe12272-bib-0030] Mackenzie, D.I. & Kendall, W.L. (2002) How should detection probability be incorporated into estimates of relative abundance? Ecology, 83, 2387–2393.

[jpe12272-bib-0031] MacKenzie, D.I. & Royle, J.A. (2005) Designing occupancy studies: general advice and allocating survey effort. Journal of Applied Ecology, 42, 1105–1114.

[jpe12272-bib-0032] Mackenzie, D.I. , Nichols, J.D. , Lachman, G.B. , Droege, S. , Royle, J.A. & Langtimm, C.A. (2002) Estimating site occupancy rates when detection probabilities are less than one. Ecology, 83, 2248–2255.

[jpe12272-bib-0033] Mackenzie, D.I. , Nichols, J.D. , Royle, J.A. , Pollock, K.H. , Bailey, L.L. & Hines, J.E. (2006) Occupancy Estimation and Modeling: Inferring Patterns and Dynamics of Species Occurrences. Elsevier Academic Press, San Diego, USA.

[jpe12272-bib-0034] Metzger, J.P. (2009) Conservation issues in the Brazilian Atlantic forest. Biological Conservation, 142, 1138–1140.

[jpe12272-bib-0035] Millennium Ecosystem Assessment (2005) Ecosystems and Human Well‐Being: Biodiversity Synthesis. World Resources Institute, Washington.

[jpe12272-bib-0036] Otis, D.L. , Burnham, K.P. , White, G.C. & Anderson, D.R. (1978) Statistical inference from capture data on closed animal populations. Wildlife Monographs, 62, 5–135.

[jpe12272-bib-0037] Pardini, R. , Bueno, A.D.A. , Gardner, T.A. , Prado, P.I. & Metzger, J.P. (2010) Beyond the fragmentation threshold hypothesis: regime shifts in biodiversity across fragmented landscapes. PLoS ONE, 5, e13666. 21060870 10.1371/journal.pone.0013666 PMC2965145

[jpe12272-bib-0038] Püttker, T. , Bueno, A.A. , de Barros, C.D. , Sommer, S. & Pardini, R. (2011) Immigration rates in fragmented landscapes ‐ empirical evidence for the importance of habitat amount for species persistence. PLoS ONE, 6, e27963. 22125643 10.1371/journal.pone.0027963 PMC3220714

[jpe12272-bib-0039] Püttker, T. , Bueno, A.A. , de Barros, C.D. , Sommer, S. & Pardini, R. (2013) Habitat specialization interacts with habitat amount to determine dispersal success of rodents in fragmented landscapes. Journal of Mammalogy, 94, 714–726.

[jpe12272-bib-0040] Rabinowitz, D. (1981) Seven Forms of Rarity. The Biological Aspects of Rare Plant Conservation. John Wiley & Sons, Chichester.

[jpe12272-bib-0041] Ramage, B.S. , Sheil, D. , Salim, H.M.W. , Fletcher, C. , Mustafa, N.Z. , Luruthusamay, J.C. *et al* (2012) Pseudoreplication in tropical forests and the resulting effects on biodiversity conservation. Conservation Biology, 27, 364–372. 10.1111/cobi.12004 23282082

[jpe12272-bib-0042] Regan, T.J. , Chades, I. & Possingham, H.P. (2011) Optimally managing under imperfect detection: a method for plant invasions. Journal of Applied Ecology, 48, 76–85.

[jpe12272-bib-0043] Regan, H.M. , Ben‐Haim, Y. , Langford, B. , Wilson, W.G. , Lundberg, P. , Andelman, S.J. & Burgman, M.A. (2005) Robust decision‐making under severe uncertainty for conservation management. Ecological Applications, 15, 1471–1477.

[jpe12272-bib-0044] Rotella, J.J. , Link, W.A. , Nichols, J.D. , Hadley, G.L. , Garrott, R.A. & Proffitt, K.M. (2009) An evaluation of density‐dependent and density‐independent influences on population growth rates in Weddell seals. Ecology, 90, 975–984. 19449692 10.1890/08-0971.1

[jpe12272-bib-0045] Royle, J.A. & Nichols, J.D. (2003) Estimating abundance from repeated presence‐absence data or point counts. Ecology, 84, 777–790.

[jpe12272-bib-0046] Ruiz‐Gutiérrez, V. , Zipkin, E.F. & Dhondt, A.A. (2010) Occupancy dynamics in a tropical bird community: unexpectedly high forest use by birds classified as non‐forest species. Journal of Applied Ecology, 47, 621–630.

[jpe12272-bib-0047] Stotz, D. , Fitzpatrick, J.W. , Parker, T.A. III & Moskovits, D.K. (1996) Neotropical Birds: Ecology and Conservation. University of Chicago Press, Chicago.

[jpe12272-bib-0048] Tingley, M.W. & Beissinger, S.R. (2012) Cryptic loss of montane avian richness and high community turnover over 100 years. Ecology, 94, 598–609. 10.1890/12-0928.1 23687886

[jpe12272-bib-0049] Welsh, A.H. , Lindenmayer, D.B. & Donnelly, C.F. (2013) Fitting and interpreting occupancy models. PLoS ONE, 8, e52015. 23326323 10.1371/journal.pone.0052015 PMC3542396

[jpe12272-bib-0050] White, G.C. (2005) Correcting wildlife counts using detection probabilities. Wildlife Research, 32, 211–216.

[jpe12272-bib-0051] White, G.C. & Burnham, K.P. (1999) Program MARK: survival estimation from populations of marked animals. Bird Study, 46, 120–139.

[jpe12272-bib-0052] Yu, J.P. & Dobson, F.S. (2000) Seven forms of rarity in mammals. Journal of Biogeography, 27, 131–139.

[jpe12272-bib-0053] Zipkin, E.F. , Dewan, A. & Royle, J.A. (2009) Impacts of forest fragmentation on species richness: a hierarchical approach to community modelling. Journal of Applied Ecology, 46, 815–822.

