# Supplementary material for: Assessing the utility of statistical adjustments for imperfect detection in tropical conservation science
Source: J Appl Ecol. 2014 Jun 2;51(4):849–59. doi: 10.1111/1365-2664.12272 (PMC4144333; doi:10.1111/1365-2664.12272)
Supplement: Supplementary file 1 — Appendix S1. List of publications obtained from the Web of Knowledge in 2012. [file JPE-51-849-s001.doc]

**Assessing the utility of statistical adjustments for imperfect detection in tropical conservation science**

Cristina BANKS-LEITE, Renata PARDINI, Danilo BOSCOLO, Camila Righetto CASSANO, Thomas PÜTTKER, Camila Santos BARROS, Jos BARLOW

*Appendix S1*

List of publications obtained from the *Web of Knowledge* in 2012. The search involved the key-words “occupancy”, along with either “detectability” or “detection probability” in the field of Topic. Only the most cited papers were analysed (101 studies with more than 10 citations in 2012) to calculate the number of studies that had either been conducted in the temperate zone, were theoretical, or had focused on a single tropical species

*References*

Bailey,L.L., Simons, T. R. & Pollock, K. H. (2004a) Spatial and temporal variation in detection probability of plethodon salamanders using the robust capture-recapture design. *Journal of Wildlife Management* **68,** 14-24.

Bailey,L.L., Simons, T. R. & Pollock, K. H. (2004b) Estimating site occupancy and species detection probability parameters for terrestrial salamanders. *Ecological Applications* **14,** 692-702.

Bailey,L.L., Hines, J. E., Nichols, J. D. & MacKenzie, D. I. (2007) Sampling design trade-offs in occupancy studies with imperfect detection: Examples and software. *Ecological Applications* **17,** 281-290.

Ball,L.C., Doherty, P. F. & McDonald, M. W. (2005) An occupancy modeling approach to evaluating a Palm Springs ground squirrel habitat model. *Journal of Wildlife Management* **69,** 894-904.

Beavers,S.C. & Ramsey, F. L. (1998) Detectability analysis in transect surveys. *Journal of Wildlife Management* **62,** 948-957.

Bibby,C.J. & Buckland, S. T. (1987) Bias of Bird Census Results Due to Detectability Varying with Habitat. *Acta Oecologica-Oecologia Generalis* **8,** 103-112.

Borgella,R. & Gavin, T. A. (2005) Avian community dynamics in a fragmented tropical landscape. *Ecological Applications* **15,** 1062-1073.

Boulinier,T., Nichols, J. D., Hines, J. E., Sauer, J. R., Flather, C. H. & Pollock, K. H. (2001) Forest fragmentation and bird community dynamics: Inference at regional scales. *Ecology* **82,** 1159-1169.

Boulinier,T., Nichols, J. D., Sauer, J. R., Hines, J. E. & Pollock, K. H. (1998) Estimating species richness: The importance of heterogeneity in species detectability. *Ecology* **79,** 1018-1028.

Cao,Y., Hawkins, C. P., Larsen, D. P. & Van Sickle, J. (2007) Effects of sample standardization on mean species detectabilities and estimates of relative differences in species richness among assemblages. *American Naturalist* **170,** 381-395.

Cozzi,G., Mueller, C. B. & Krauss, J. (2008) How do local habitat management and landscape structure at different spatial scales affect fritillary butterfly distribution on fragmented wetlands? *Landscape Ecology* **23,** 269-283.

Cranmer,L., McCollin, D. & Ollerton, J. (2012) Landscape structure influences pollinator movements and directly affects plant reproductive success. *Oikos* **121,** 562-568.

de Solla,S.R., Shirose, L. J., Fernie, K. J., Barrett, G. C., Brousseau, C. S. & Bishop, C. A. (2005) Effect of sampling effort and species detectability on volunteer based anuran monitoring programs. *Biological Conservation* **121,** 585-594.

De Solla,S.R., Fernie, K. J., Barrett, G. C. & Bishop, C. A. (2006) Population trends and calling phenology of anuran populations surveyed in Ontario estimated using acoustic surveys. *Biodiversity and Conservation* **15,** 3481-3497.

Dettmers,R., Buehler, D. A., Bartlett, J. G. & Klaus, N. A. (1999) Influence of point count length and repeated visits on habitat model performance. *Journal of Wildlife Management* **63,** 815-823.

Devictor,V. & Jiguet, F. (2007) Community richness and stability in agricultural landscapes: The importance of surrounding habitats. *Agriculture Ecosystems & Environment* **120,** 179-184.

Devictor,V. & Robert, A. (2009) Measuring community responses to large-scale disturbance in conservation biogeography. *Diversity and Distributions* **15,** 122-130.

Dorazio,R.M., Royle, J. A., Soderstrom, B. & Glimskar, A. (2006) Estimating species richness and accumulation by modeling species occurrence and detectability. *Ecology* **87,** 842-854.

Dorazio,R.M. (2007) On the choice of statistical models for estimating occurrence and extinction from animal surveys. *Ecology* **88,** 2773-2782.

Edwards,T.C., Cutler, D. R., Geiser, L., Alegria, J. & McKenzie, D. (2004) Assessing rarity of species with low detectability: Lichens in Pacific Northwest forests. *Ecological Applications* **14,** 414-424.

Field,S.A., Tyre, A. J. & Possingham, H. P. (2005) Optimizing allocation of monitoring effort under economic and observational constraints. *Journal of Wildlife Management* **69,** 473-482.

Franklin,J., Wejnert, K. E., Hathaway, S. A., Rochester, C. J. & Fisher, R. N. (2009) Effect of species rarity on the accuracy of species distribution models for reptiles and amphibians in southern California. *Diversity and Distributions* **15,** 167-177.

Garrard,G.E., Bekessy, S. A., McCarthy, M. A. & Wintle, B. A. (2008) When have we looked hard enough? A novel method for setting minimum survey effort protocols for flora surveys. *Austral Ecology* **33,** 986-998.

Gibbs,J.P. & Melvin, S. M. (1993) Call-Response Surveys for Monitoring Breeding Waterbirds. *Journal of Wildlife Management* **57,** 27-34.

Gimenez,O., Rossi, V., Choquet, R., Dehais, C., Doris, B., Varella, H., Vila, J. P. & Pradel, R. (2007) State-space modelling of data on marked individuals. *Ecological Modelling* **206,** 431-438.

Govindan,B.N., Kéry, M. & Swihart, R. K. (2012) Host selection and responses to forest fragmentation in acorn weevils: inferences from dynamic occupancy models. *Oikos* **121,** 623-633.

Grand,J., Cummings, M. P., Rebelo, T. G., Ricketts, T. H. & Neel, M. C. (2007) Biased data reduce efficiency and effectiveness of conservation reserve networks. *Ecology Letters* **10,** 364-374.

Gu,W.D. & Swihart, R. K. (2004) Absent or undetected? Effects of non-detection of species occurrence on wildlife-habitat models. *Biological Conservation* **116,** 195-203.

Gutzwiller,K.J., Marcum, H. A., Harvey, H. B., Roth, J. D. & Anderson, S. H. (1998) Bird tolerance to human intrusion in Wyoming montane forests. *Condor* **100,** 519-527.

Heard,S.B. (1998) Resource patch density and larval aggregation in mushroom-breeding flies. *Oikos* **81,** 187-195.

Hines,J., Nichols, J., Royle, J., MacKenzie, D., I, Gopalaswamy, A., Kumar, N. & Karanth, K. (2010) Tigers on trails: occupancy modeling for cluster sampling. *Ecological Applications* **20,** 1456-1466.

Hossack,B.R. & Corn, P. S. (2007) Responses of pond-breeding amphibians to wildfire: Short-term patterns in occupancy and colonization. *Ecological Applications* **17,** 1403-1410.

Hyde,E.J. & Simons, T. R. (2001) Sampling plethodontid salamanders: Sources of variability. *Journal of Wildlife Management* **65,** 624-632.

Johns,A.D. (1985) Differential Detectability of Primates Between Primary and Selectively Logged Habitats and Implications for Population Surveys. *American Journal of Primatology* **8,** 31-36.

Johnson,D.H. (2008) In Defense of indices: The case of bird surveys. *Journal of Wildlife Management* **72,** 857-868.

Joseph,L.N., Field, S. A., Wilcox, C. & Possingham, H. P. (2006) Presence-absence versus abundance data for monitoring threatened species. *Conservation Biology* **20,** 1679-1687.

Karanth,K.U., Nichols, J. D., Kumar, N. S., Link, W. A. & Hines, J. E. (2004) Tigers and their prey: Predicting carnivore densities from prey abundance. *Proceedings of the National Academy of Sciences of the United States of America* **101,** 4854-4858.

Karanth,K.K., Nichols, J. D., Hines, J. E., Karanth, K. & Christensen, N. L. (2009) Patterns and determinants of mammal species occurrence in India. *Journal of Applied Ecology* **46,** 1189-1200.

Kendall,W.L. & White, G. C. (2009) A cautionary note on substituting spatial subunits for repeated temporal sampling in studies of site occupancy. *Journal of Applied Ecology* **46,** 1182-1188.

Kery,M. (2004) Extinction rate. estimates for plant populations in revisitation studies: Importance of detectability. *Conservation Biology* **18,** 570-574.

Kery,M. & Gregg, K. B. (2003) Effects of life-state on detectability in a demographic study of the terrestrial orchid Cleistes bifaria. *Journal of Ecology* **91,** 265-273.

Kery,M., Royle, J. A. & Schmid, H. (2005) Modeling avian abundance from replicated counts using binomial mixture models. *Ecological Applications* **15,** 1450-1461.

Kery,M. & Schmid, H. (2004) Monitoring programs need to take into account imperfect species detectability. *Basic and Applied Ecology* **5,** 65-73.

Kery,M. & Schmid, H. (2006) Estimating species richness: calibrating a large avian monitoring programme. *Journal of Applied Ecology* **43,** 101-110.

Kery,M. (2008) Estimating abundance from bird counts: Binomial mixture models uncover complex covariate relationships. *Auk* **125,** 336-345.

Kery,M., Dorazio, R. M., Soldaat, L., van Strien, A., Zuiderwijk, A. & Royle, J. (2009a) Trend estimation in populations with imperfect detection. *Journal of Applied Ecology* **46,** 1163-1172.

Kery,M. & Plattner, M. (2007) Species richness estimation and determinants of species detectability in butterfly monitoring programmes. *Ecological Entomology* **32,** 53-61.

Kery,M., Royle, J., Plattner, M. & Dorazio, R. M. (2009b) Species richness and occupancy estimation in communities subject to temporary emigration. *Ecology* **90,** 1279-1290.

Kery,M., Spillmann, J. H., Truong, C. & Holderegger, R. (2006) How biased are estimates of extinction probability in revisitation studies? *Journal of Ecology* **94,** 980-986.

Kroll,A.J., Risenhoover, K., McBride, T., Beach, E., Kernohan, B. J., Light, J. & Bach, J. (2008) Factors influencing stream occupancy and detection probability parameters of stream-associated amphibians in commercial forests of Oregon and Washington, USA. *Forest Ecology and Management* **255,** 3726-3735.

Larrucea,E.S., Brussard, P. F., Jaeger, M. M. & Barrett, R. H. (2007) Cameras, coyotes, and the assumption of equal detectability. *Journal of Wildlife Management* **71,** 1682-1689.

Leal,M. & Fleishman, L. J. (2004) Differences in visual signal design and detectability between allopatric populations of Anolis lizards. *American Naturalist* **163,** 26-39.

Link,W.A. & Sauer, J. R. (2002) A hierarchical analysis of population change with application to Cerulean Warblers. *Ecology* **83,** 2832-2840.

Linkie,M., Chapron, G., Martyr, D. J., Holden, J. & Leader-Williams, N. (2006) Assessing the viability of tiger subpopulations in a fragmented landscape. *Journal of Applied Ecology* **43,** 576-586.

Luiselli,L. (2006) Site occupancy and density of sympatric Gaboon viper (Bitis gabonica) and nose-horned viper (Bitis nasicornis). *Journal of Tropical Ecology* **22,** 555-564.

Lynam,A.J., Rabinowitz, A., Myint, T., Maung, M., Latt, K. T. & Po, S. H. (2009) Estimating abundance with sparse data: tigers in northern Myanmar. *Population Ecology* **51,** 115-121.

MacKenzie,D., I & Nichols, J. (2004) Occupancy as a surrogate for abundance estimation. *Animal Biodiversity and Conservation* **27,** 461-467.

MacKenzie,D.I. (2006) Modeling the probability of resource use: The effect of, and dealing with, detecting a species imperfectly. *Journal of Wildlife Management* **70,** 367-374.

MacKenzie,D.I. (2005) What are the issues with presence-absence data for wildlife managers? *Journal of Wildlife Management* **69,** 849-860.

MacKenzie,D.I., Bailey, L. L. & Nichols, J. D. (2004) Investigating species co-occurrence patterns when species are detected imperfectly. *Journal of Animal Ecology* **73,** 546-555.

MacKenzie,D.I., Nichols, J. D., Hines, J. E., Knutson, M. G. & Franklin, A. B. (2003) Estimating site occupancy, colonization, and local extinction when a species is detected imperfectly. *Ecology* **84,** 2200-2207.

MacKenzie,D.I., Nichols, J. D., Lachman, G. B., Droege, S., Royle, J. A. & Langtimm, C. A. (2002) Estimating site occupancy rates when detection probabilities are less than one. *Ecology* **83,** 2248-2255.

MacKenzie,D.I., Nichols, J. D., Sutton, N., Kawanishi, K. & Bailey, L. L. (2005) Improving inferences in popoulation studies of rare species that are detected imperfectly. *Ecology* **86,** 1101-1113.

MacKenzie,D.I. & Royle, J. A. (2005) Designing occupancy studies: general advice and allocating survey effort. *Journal of Applied Ecology* **42,** 1105-1114.

Marsden,S.J. (1999) Estimation of parrot and hornbill densities using a point count distance sampling method. *Ibis* **141,** 377-390.

Marsh,D.M. & Beckman, N. G. (2004) Effects of forest roads on the abundance and activity of terrestrial salamanders. *Ecological Applications* **14,** 1882-1891.

Martin,J., Kitchens, W. M. & Hines, J. E. (2007) Importance of well-designed monitoring programs for the conservation of endangered species: Case study of the snail kite. *Conservation Biology* **21,** 472-481.

Mazerolle,M.J., Desrochers, A. & Rochefort, L. (2005) Landscape characteristics influence pond occupancy by frogs after accounting for detectability. *Ecological Applications* **15,** 824-834.

Mazerolle,M.J., Bailey, L. L., Kendall, W. L., Royle, J., Converse, S. J. & Nichols, J. D. (2007) Making great leaps forward: Accounting for detectability in herpetological field studies. *Journal of Herpetology* **41,** 672-689.

Moore,J.E., Scheiman, D. M. & Swihart, R. K. (2004) Field comparison of removal and modified double-observer modeling for estimating detectability and abundance of birds. *Auk* **121,** 865-876.

Moore,J.E. & Swihart, R. K. (2005) Modeling patch occupancy by forest rodents: Incorporating detectability and spatial autocorrelation with hierarchically structured data. *Journal of Wildlife Management* **69,** 933-949.

Moritz,C., Patton, J. L., Conroy, C. J., Parra, J. L., White, G. C. & Beissinger, S. R. (2008) Impact of a century of climate change on small-mammal communities in Yosemite National Park, USA. *Science* **322,** 261-264.

Newson,S.E., Evans, K. L., Noble, D. G., Greenwood, J. J. & Gaston, K. J. (2008) Use of distance sampling to improve estimates of national population sizes for common and widespread breeding birds in the UK. *Journal of Applied Ecology* **45,** 1330-1338.

Nichols,J.D., Bailey, L. L., O'Connell, A. F., Talancy, N. W., Grant, E. H., Gilbert, A. T., Annand, E. M., Husband, T. P. & Hines, J. E. (2008) Multi-scale occupancy estimation and modelling using multiple detection methods. *Journal of Applied Ecology* **45,** 1321-1329.

O'Connell,A.F., Talancy, N. W., Bailey, L. L., Sauer, J. R., Cook, R. & Gilbert, A. T. (2006) Estimating site occupancy and detection probability parameters for meso- and large mammals in a coastal ecosystem. *Journal of Wildlife Management* **70,** 1625-1633.

Olson,G.S., Anthony, R. G., Forsman, E. D., Ackers, S. H., Loschl, P. J., Reid, J. A., Dugger, K. M., Glenn, E. M. & Ripple, W. J. (2005) Modeling of site occupancy dynamics for northern spotted owls, with emphasis on the effects of barred owls. *Journal of Wildlife Management* **69,** 918-932.

Pellet,J. & Schmidt, B. R. (2005) Monitoring distributions using call surveys: estimating site occupancy, detection probabilities and inferring absence. *Biological Conservation* **123,** 27-35.

Pellet,J. (2008) Seasonal variation in detectability of butterflies surveyed with Pollard walks. *Journal of Insect Conservation* **12,** 155-162.

Pellet,J., Fleishman, E., Dobkin, D. S., Gander, A. & Murphy, D. D. (2007) An empirical evaluation of the area and isolation paradigm of metapopulation dynamics. *Biological Conservation* **136,** 483-495.

Peterson,J.T. & Dunham, J. (2003) Combining inferences from models of capture efficiency, detectability, and suitable habitat to classify landscapes for conservation of threatened bull trout. *Conservation Biology* **17,** 1070-1077.

Pollock,K.H., Marsh, H. D., Lawler, I. R. & Alldredge, M. W. (2006) Estimating animal abundance in heterogeneous environments: An application to aerial surveys for Dugongs. *Journal of Wildlife Management* **70,** 255-262.

Rota,C.T., Fletcher, R. J., Dorazio, R. M. & Betts, M. G. (2009) Occupancy estimation and the closure assumption. *Journal of Applied Ecology* **46,** 1173-1181.

Royle,J. & Dorazio, R. M. (2006) Hierarchical models of animal abundance and occurrence. *Journal of Agricultural Biological and Environmental Statistics* **11,** 249-263.

Royle,J. & Kery, M. (2007) A Bayesian state-space formulation of dynamic occupancy models. *Ecology* **88,** 1813-1823.

Royle,J., Kery, M., Gautier, R. & Schmid, H. (2007) Hierarchical spatial models of abundance and occurrence from imperfect survey data. *Ecological Monographs* **77,** 465-481.

Royle,J.A. (2004) Modeling abundance index data from anuran calling surveys. *Conservation Biology* **18,** 1378-1385.

Royle,J.A. (2006) Site occupancy models with heterogeneous detection probabilities. *Biometrics* **62,** 97-102.

Royle,J.A. & Link, W. A. (2005) A general class of multinomial mixture models for anuran calling survey data. *Ecology* **86,** 2505-2512.

Royle,J.A. & Nichols, J. D. (2003) Estimating abundance from repeated presence-absence data or point counts. *Ecology* **84,** 777-790.

Royle,J.A., Nichols, J. D. & Kery, M. (2005) Modelling occurrence and abundance of species when detection is imperfect. *Oikos* **110,** 353-359.

Russell,R.E., Royle, J., Saab, V. A., Lehmkuhl, J. F., Block, W. M. & Sauer, J. R. (2009) Modeling the effects of environmental disturbance on wildlife communities: avian responses to prescribed fire. *Ecological Applications* **19,** 1253-1263.

Skorupa,J.P. (1987) Do Line-Transect Surveys Systematically Underestimate Primate Densities in Logged Forests. *American Journal of Primatology* **13,** 1-9.

Stanley,T.R. & Royle, J. A. (2005) Estimating site occupancy and abundance using indirect detection indices. *Journal of Wildlife Management* **69,** 874-883.

Thiollay,J.-M. (1989) Censusing of Diurnal Raptors in A Primary Rain Forest Comparative Methods and Species Detectability. *Journal of Raptor Research* **23,** 72-84.

Varman,K.S. & Sukumar, R. (1995) The Line Transect Method for Estimating Densities of Large Mammals in A Tropical Deciduous Forest - An Evaluation of Models and Field Experiments. *Journal of Biosciences* **20,** 273-287.

Watson,J.W., Hays, D. W. & Pierce, D. J. (1999) Efficacy of northern goshawk broadcast surveys in Washington state. *Journal of Wildlife Management* **63,** 98-106.

Weir,L.A., Royle, J. A., Nanjappa, P. & Jung, R. E. (2005) Modeling anuran detection and site occupancy on North American Amphibian Monitoring Program (NAAMP) routes in Maryland. *Journal of Herpetology* **39,** 627-639.

Wenger,S.J. & Freeman, M. C. (2008) Estimating Species Occurrence, Abundance, and Detection Probability Using Zero-Inflated Distributions. *Ecology* **89,** 2953-2959.

Wintle,B.A., Kavanagh, R. P., McCarthy, M. A. & Burgman, M. A. (2005) Estimating and dealing with detectability in occupancy surveys for forest owls and arboreal marsupials. *Journal of Wildlife Management* **69,** 905-917.

Wintle,B.A., McCarthy, M. A., Parris, K. M. & Burgman, M. A. (2004) Precision and bias of methods for estimating point survey detection probabilities. *Ecological Applications* **14,** 703-712.

Zipkin,E.F., DeWan, A. & Royle, J. (2009) Impacts of forest fragmentation on species richness: a hierarchical approach to community modelling. *Journal of Applied Ecology* **46,** 815-822.
