# Supplementary material for: Assessing the utility of statistical adjustments for imperfect detection in tropical conservation science
Source: J Appl Ecol. 2014 Jun 2;51(4):849–59. doi: 10.1111/1365-2664.12272 (PMC4144333; doi:10.1111/1365-2664.12272)

**Assessing the utility of statistical adjustments for imperfect detection in tropical conservation science**

Cristina BANKS-LEITE, Renata PARDINI, Danilo BOSCOLO, Camila Righetto CASSANO, Thomas PÜTTKER, Camila Santos BARROS, Jos BARLOW

*Appendix S2*

#Model codes used to calculate species occupancy at the landscapes with 10, 30, 50 and 90 forest cover, while accounting for changes in detectability due to sampling effort and patch size. For more details about the model see Zipkin, E.F., Dewan, A. & Royle, J. A. (2009) Impacts of forest fragmentation on species richness: a hierarchical approach to community modelling. Journal of Applied Ecology 46, 815-822.

model {

#Prior distributions on the community level occupancy and detection covariates

psi.mean ~ dunif(0,1)

a <- log(psi.mean) - log(1-psi.mean)

theta.mean ~ dunif(0,1)

b <- log(theta.mean) - log(1-theta.mean)

for(f in 1:4){

mu.alpha[f] ~ dnorm(0, 0.001)}

for(l in 1:2){

beta[l] ~ dnorm(0, 0.001)}

for(g in 1:6){

sigma[g] ~ dunif(0,3)

tau[g] <- 1/(sigma[g] * sigma[g])}

for(i in S1:S2){

#Prior distributions for the occupancy and detection covariates for each species

u[i] ~ dnorm(a, tau[1])

v[i] ~ dnorm(b, tau[2])

alpha1[i] ~ dnorm(mu.alpha[1], tau[3])

alpha2[i] ~ dnorm(mu.alpha[2], tau[4])

alpha3[i] ~ dnorm(mu.alpha[3], tau[5])

alpha4[i] ~ dnorm(mu.alpha[4], tau[6])

#Estimate the occupancy probability (latent Z matrix) for each species at each landscape

for(j in 1:J){

logit(psi[j,i]) <- u[i] + alpha1[i]*lands[j,1] + alpha2[i]*lands[j,2] + alpha3[i]*lands[j,3] + alpha4[i]*lands[j,4]

Z[j,i] ~ dbin(psi[j,i], 1)

#Estimate the species specific detection probability

for(k in 1:K[j]){

logit(theta[j,k,i]) <- v[i] + beta[1]*effort[j,k] + beta[2]*area[j]

mu.theta[j,k,i] <- theta[j,k,i]*Z[j,i]

X[j,k,i] ~ dbin(mu.theta[j,k,i], 1)

Xnew[j,k,i] ~ dbin(mu.theta[j,k,i], 1)

#Create simulated dataset to calculate the Bayesian p-value

d[j,k,i]<- abs(X[j,k,i] - mu.theta[j,k,i])

dnew[j,k,i]<- abs(Xnew[j,k,i] - mu.theta[j,k,i])

d2[j,k,i]<- pow(d[j,k,i],2)

dnew2[j,k,i]<- pow(dnew[j,k,i],2)

}

dsum[j,i]<- sum(d2[j,1:K[j],i])

dnewsum[j,i]<- sum(dnew2[j,1:K[j],i])

}

}

#Calculate the discrepancy measure, which is then defined as the mean(p.fit > p.fitnew). If p.fit == p.fitnew then model is good.

p.fit<-sum(dsum[1:J,S1:S2])

p.fitnew<-sum(dnewsum[1:J,S1:S2])

}

**Figure S1**: Land-use map of (a) the cacao growing region of southern Bahia and (b) the study region of large mammal survey showing the sampling sites.


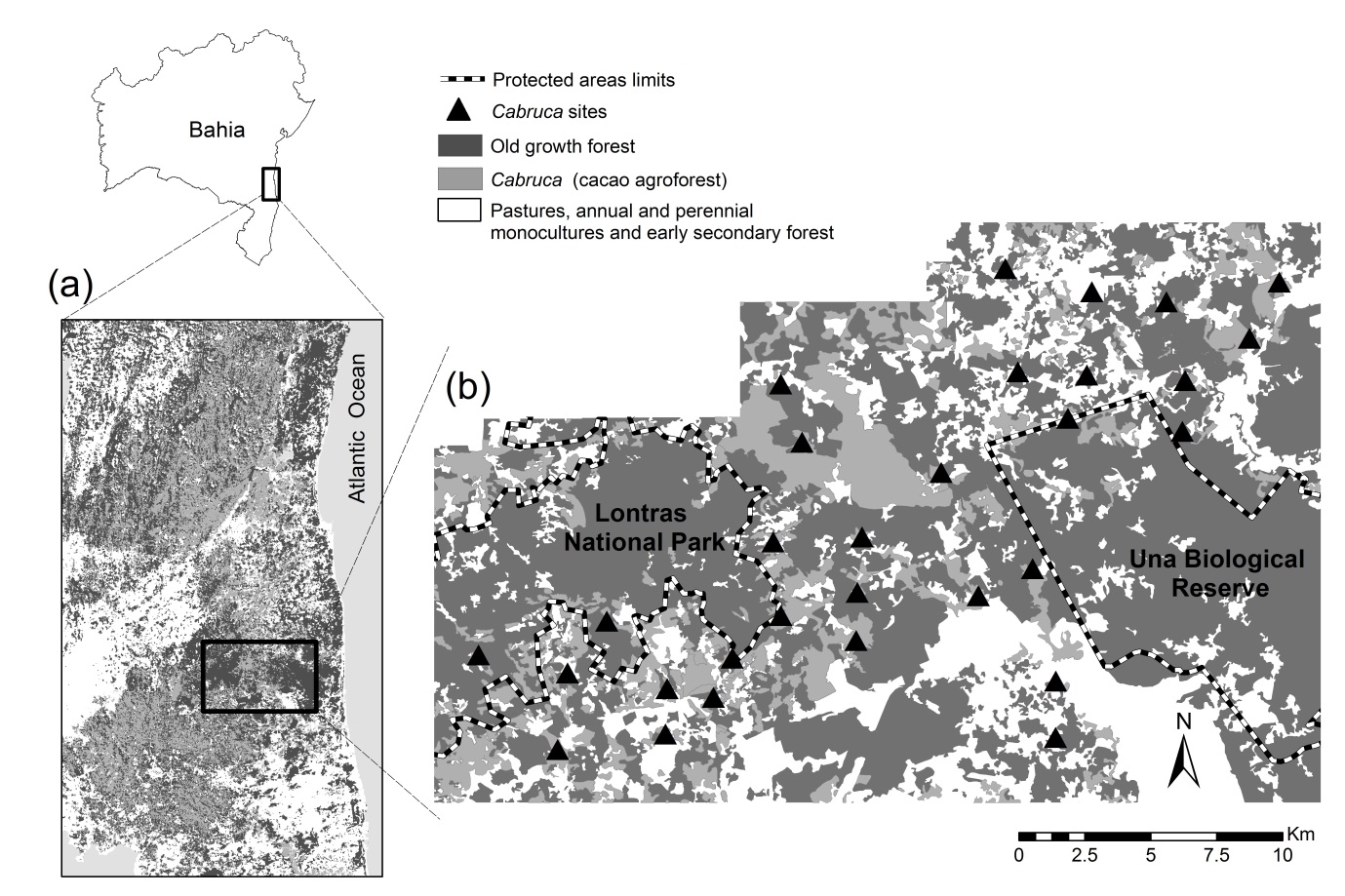


**Figure S2** - Map of the study area in the state of São Paulo, Brazil, showing the location of the fragmented landscapes (dotted lines) and adjacent continuous forest, and the sampling sites where understory birds were captured (circles). Panel A depicts the 10% FC landscape, panel B – 30% FC landscape, panel C – 50% FC landscape. Figure redrawn from Banks-Leite C., Ewers R.M., Kapos V., Martensen A.C. & Metzger J.P. (2011). Comparing species and measures of landscape structure as indicators of conservation importance. Journal of Applied Ecology, 48, 706-714.


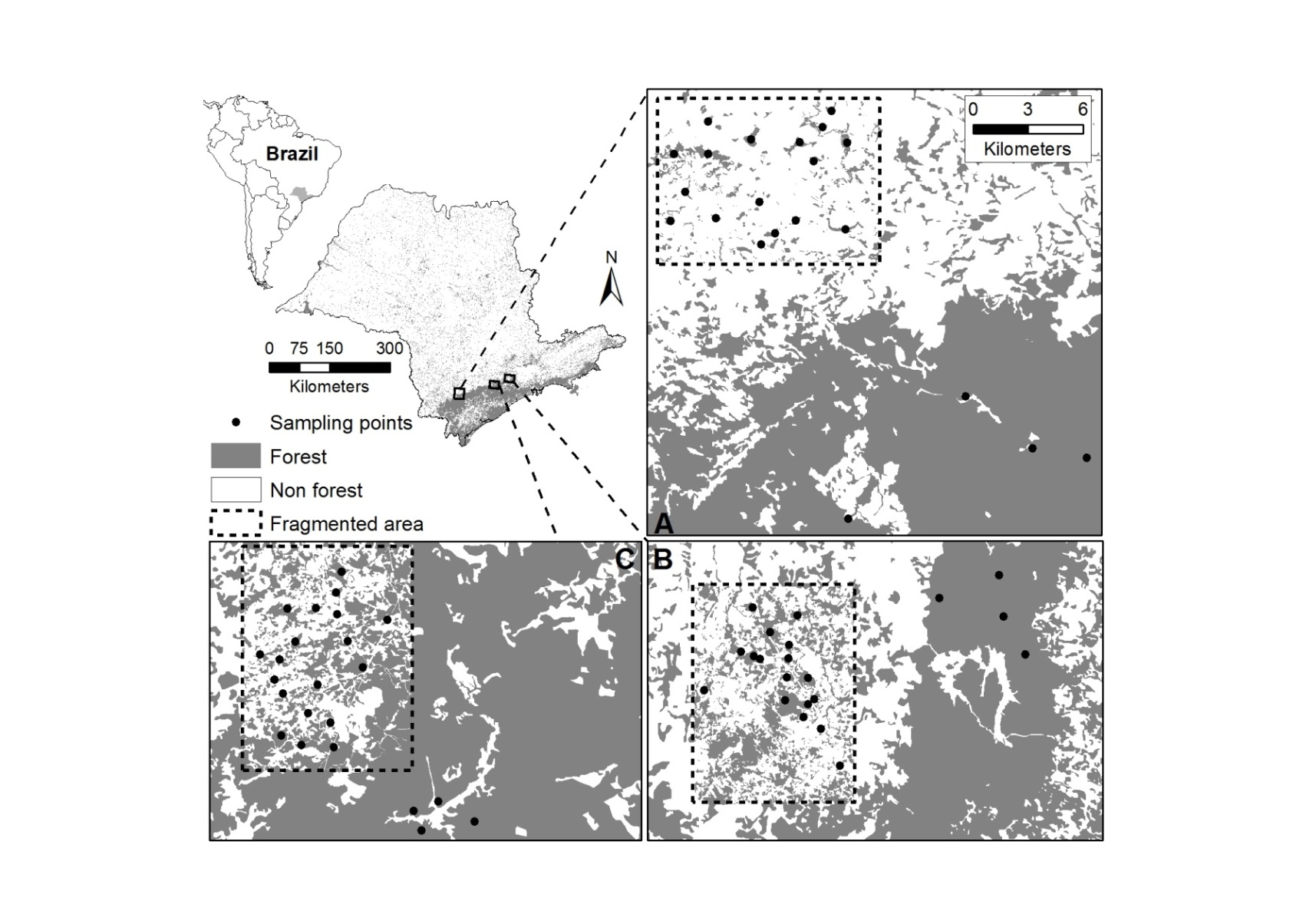

Supplement: Supplementary file 2 — Appendix S2. Model codes used to calculate species occupancy at the landscapes with 10, 30, 50 and 90 forest cover, while accounting for changes in detectability due to sampling effort and patch size. [file JPE-51-849-s002.doc]
